# A Novel Pan-Genome Reverse Vaccinology Approach Employing a Negative-Selection Strategy for Screening Surface-Exposed Antigens against *leptospirosis*

**DOI:** 10.3389/fmicb.2017.00396

**Published:** 2017-03-14

**Authors:** LingBing Zeng, Dongliang Wang, NiYa Hu, Qing Zhu, Kaishen Chen, Ke Dong, Yan Zhang, YuFeng Yao, XiaoKui Guo, Yung-Fu Chang, YongZhang Zhu

**Affiliations:** ^1^Department of Laboratory Medicine, the First Affiliated Hospital of NanChang UniversityNanchang, China; ^2^Department of Medical Microbiology and Immunology, Shanghai Jiao Tong University School of MedicineShanghai, China; ^3^CAS Key Laboratory for Biological Effects of Nanomaterials and Nanosafety, National Center for Nanoscience and TechnologyBeijing, China; ^4^Deparment of Molecular Immunology, Institute of Medical Biology, Chinese Academy of Medical Sciences, Peking Union Medical CollegeKunming, China; ^5^Department of Population Medicine and Diagnostic Sciences, College of Veterinary Medicine, Cornell UniversityIthaca, NY, USA

**Keywords:** reverse vaccinology (RV), negative selection strategy, surface-exposed proteins, vaccine candidate, *L. interrogans*

## Abstract

Reverse vaccinology (RV) has been widely used for screening of surface-exposed proteins (PSEs) of important pathogens, including outer membrane proteins (OMPs), and extracellular proteins (ECPs) as potential vaccine candidates. In this study, we applied a novel RV negative strategy and a pan-genome analysis for screening of PSEs from 17 *L. interrogans* strains covering 11 predominately epidemic serovars and 17 multilocus typing (MLST) sequence types (STs) worldwide. Our results showed, for instance, out of a total of 633 predicted PSEs in strain 56601, 92.8% were OMPs or ECPs (588/633). Among the 17 strains, 190 core PSEs, 913 dispensable PSEs and 861 unique PSEs were identified. Of the 190 PSEs, 121 were further predicted to be highly antigenic and thus may serve as potential vaccine candidates against leptospirosis. With the exception of LipL45, OmpL1, and LigB, the majority of the 121 PSEs were newly identified antigens. For example, hypothetical proteins BatC, LipL71, and the OmpA family proteins sharing many common features, such as surface-exposed localization, universal conservation, and eliciting strong antibody responses in patients, are regarded as the most promising vaccine antigens. Additionally, a wide array of potential virulence factors among the predicted PSEs including TonB-dependent receptor, sphingomyelinase 2, leucine-rich repeat protein, and 4 neighboring hypothetical proteins were identified as potential antigenicity, and deserve further investigation. Our results can contribute to the prediction of suitable antigens as potential vaccine candidates against leptospirosis and also provide further insights into mechanisms of leptospiral pathogenicity. In addition, our novel negative-screening strategy combined with pan-genome analysis can be a routine RV method applied to numerous other pathogens.

## Introduction

Leptospirosis, caused by pathogenic spirochete bacteria of the genus *Leptospira*, is one of the most common zoonotic diseases worldwide. Leptospirosis has been recognized as an emerging disease with more than half a million patients reported annually (Adler et al., 2011). Pathogenic *Leptospira* spp. are transmitted mainly by direct contact with infected animals or by exposure to water or soil contaminated by the urine of infected animals (Faine, 1994). To date, more than 250 serovars have been observed in pathogenic *Leptospira* (Zhang et al., 2012). At the present time, available *leptospira* vaccines are inactivated whole cell products that provide inadequate protection against most serovars and cannot provide cross-protection against a large number of serogroups of pathogenic leptospires (Faisal et al., 2008). Therefore, there is an urgent need to develop a long-term and cross-protective vaccine set against leptospirosis.

A revolutionary vaccine research strategy, reverse vaccinology (RV), was able to identify five suitable serogroup B meningococcal vaccine candidates (Pizza et al., 2000). Subsequently, RV has been widely applied to a wide range of bacterial pathogens, including *Streptococcus pneumoniae, S. agalactiae, Staphylococcus aureus, Porphyromonas gingivalis, Chlamydia pneumonia*, and *L. interrogans* (Paton and Giammarinaro, 2001; Wizemann et al., 2001; Hava and Camilli, 2002; Gamberini et al., 2005; Maione et al., 2005; Mora et al., 2005; Tettelin et al., 2005; Falugi et al., 2008; Seib et al., 2012). Generally, Gram-negative bacteria have five subcellular location sites including cytoplasm, inner membrane, outer membrane, periplasm, and extracellular space. According to RV theory, except for cytoplasmic and inner membrane proteins, proteins located in the other sites can be regarded as PSEs, and are the most suitable vaccine candidates due to their high susceptibility to antibody recognition and eliciting protective immune responses. The *in silico* approach of RV is a novel and integrative method that uses available bioinformatic tools in the first step of vaccine development. The currently used *in silico* strategy of RV is to focus only on OMPs and ECPs positively predicted by several bioinformatic tools, such as PSORTb, Cello, and P-classifier. This approach may overlook numerous unknown proteins as potential vaccine candidates because a relatively high proportion of proteins are not covered by these bioinformatic tools. For instance, the most frequently used tool, PSORTb, achieved the greatest degree of precision, but as many as 30.8% (1,140) of *str.56601* proteins were not selected as potential vaccine candidates for further screening, simply due to the fact that the localization sites of these proteins were unknown. This is illustrated by the extracellular virulence factor of *Bordetella pertussis*-pertussis toxin, the only indispensable component of acellular pertussis vaccines, which was predicted as an “unknown” protein by PSORTB. Furthermore, OMPs, ECPs, and Periplasmic proteins (PMs) were predicted far less accurately and reliably than cytoplasmic proteins (CYTs) and inner membrane proteins (IMPs) by these frequently used bioinformatic tools, including PSORTb, Cello, Proteome Analysis, Subloc, and LOCtree (Gardy and Brinkman, 2006). The usage of these popular bioinformatic tools remains a matter for further investigation, as they may miss or exclude highly antigenic vaccine candidates. Here, in this study, a novel RV prediction method employing a negative selection strategy was developed to reliably identify potential vaccine candidates by removing CYTs and IMPs. Based on our novel RV strategy, these “unknown” proteins that are further predicted as CYTs or IMPs by multiple tools according to our criteria are excluded, and the remaining “unknown” proteins, which might be surface-exposed, are retained in the final vaccine candidates list for further screening. Thus, we can greatly reduce the risk of missing potential vaccine candidates among these “unknown” proteins predicted by one of these computational methods.

Early RV efforts were focused mainly on a single genome of a pathogenic strain or species. This limited focus renders it impossible to develop a universal vaccine comprising biologically cross-protective antigens against multiple serovars, strains, or pathovars of one pathogen. To alleviate this shortcoming, pan-genome strategies have been developed to identify potential cross-protective antigens using multiple genomes of the same species, such as group B *Streptococcus* spp. (Maione et al., 2005).

In this study, we have applied a new *in silico* RV negative selection strategy combining a pan-genome analysis to screen PSEs as vaccine candidates to provide a framework for future vaccine development against leptospirosis. In addition, potential virulence factors of leptospira were also further analyzed in this study. Future efforts will be targeted toward the experimental characterization of these identified PSEs in our study, as well as screening their potential as vaccine candidates in an animal model.

## Materials and methods

### Selection of leptospiral genome sequences

Information for leptospiral serovars and multilocus sequence typing were combined to select suitable strains of *L. interrogans*. Finally, the 17 representative *L. interrogans* strains covering 11 dominantly epidemic serovars and 17 MLST sequence types (STs) worldwide were selected. For instance, more than 90% of Chinese epidemic or outbreak strains belonged to the 11 dominant serovars (Zhang et al., 2012). The proteomes of all strains were downloaded from the Pathosystems Resource Integration Center (PATRIC) website (www.patricbrc.org) and detailed information about the selected strains is presented in Table 1.

**Table 1:** **All information of the 17 representative strains of pathogenic *L. interrogans* used in this study**.

**Strains**	**MLST**	**Serovar**	**Isolated location**	**Host**	**Contig**
*Leptospirainterrogans*serovar Australia str. 2002000624	ST51	Australia	Hawaii, USA	Human	146
*Leptospirainterrogans*serovarBataviae str. 2006006976	ST50	Bataviae	Egypt	Human	314
*Leptospirainterrogans*serovarBataviae str. L1111	ST42	Bataviae	Thailand	Human	157
*Leptospirainterrogans*serovarBataviae str. UI 08561	ST79	Bataviae	Laos	Human	300
*Leptospirainterrogans*serovarBulgarica str. Mallika	ST112	Bulgarica	India	Human	335
*Leptospirainterrogans*serovarCopenhageni str. Fiocruz L1-130	ST17	Copenhageni	Brazil	Human	2
*Leptospirainterrogans*serovarGrippotyphosa str. UI 08368	ST77	Grippotyphosa	Laos	Human	369
*Leptospirainterrogans*serovarGrippotyphosa str. UI 08434	ST82	Grippotyphosa	Laos	Human	237
*Leptospirainterrogan*sserovarGrippotyphosa str. UI 12764	ST85	Grippotyphosa	Laos	Human	147
*Leptospirainterrogans*serovar Lai str. 56601	ST1	Lai	China	Human	2
*Leptospirainterrogans*serovarManilae str. M001_Tn_Mutant_Parent	ST57	Manilae	Philippinnes	Human	271
*Leptospirainterrogans*serovarMedanensis str. UT053	ST46	Medanensis	Thailand	Human	188
*Leptospirainterrogans*serovarMuenchen str. Brem 129	ST24	Muenchen	Germany	Horse	322
*Leptospirainterrogans*serovar Pomona str. Pomona	ST37	Pomona	Australia	Human	118
*Leptospirainterrogans*serovarPyrogenes str. 2006006956	ST88	Pyrogenes	Egypt	Human	344
*Leptospirainterrogans*serovarPyrogenes str. L0374	ST49	Pyrogenes	Thailand	Human	169
*Leptospirainterrogans*serovarPyrogenes str. SriLanka1	ST75	Pyrogenes	Tanzania	Rodent, Mastomyssp	667

### Predicting strategy for PSEs of *L. interrogans*

**Figure 1:**
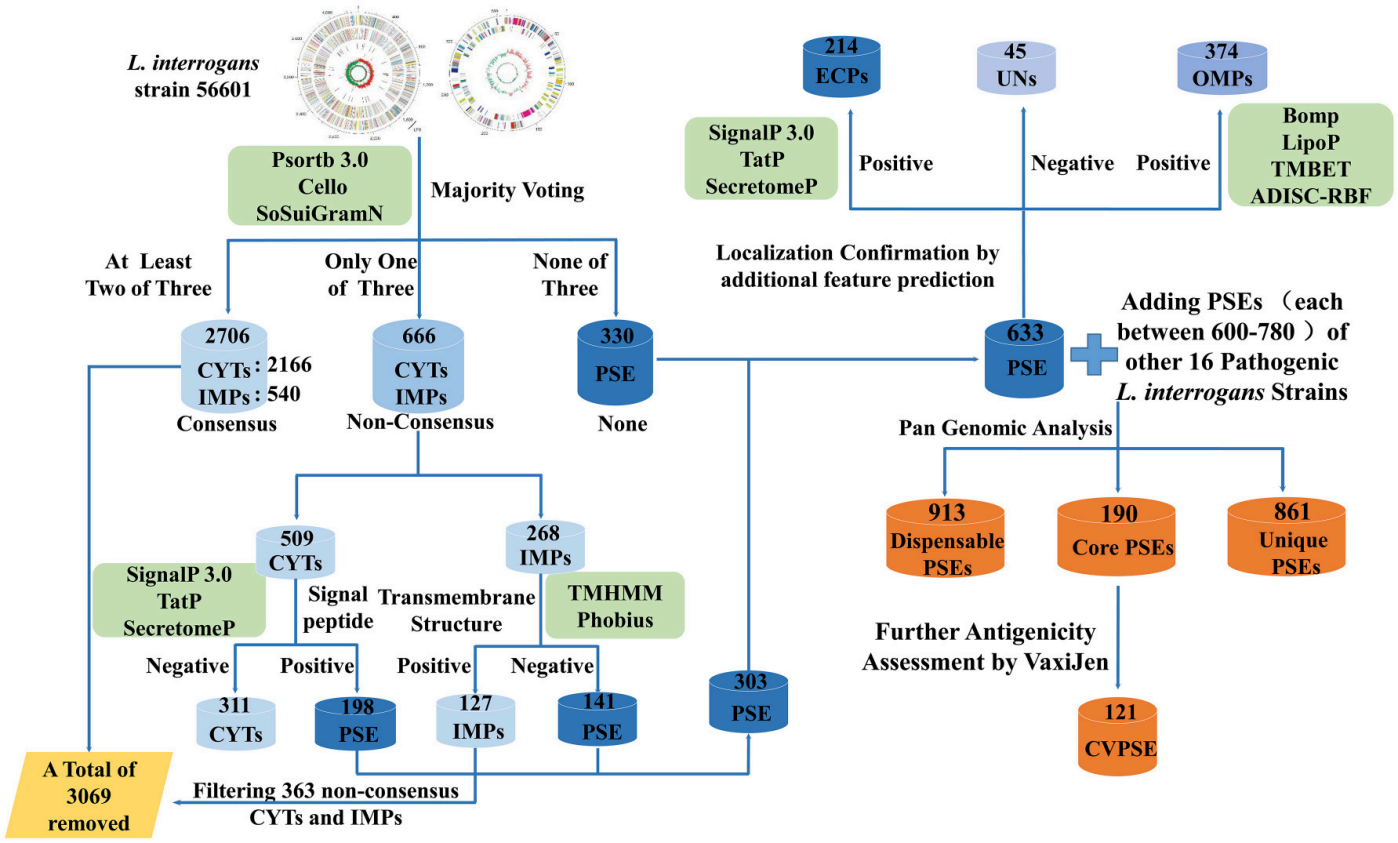
**Schematic representation of the novel strategy of reverse vaccinology applied to Pathogenic *L.interrogans***. In Figure 1, *L. interrogans str*.56601 was selected as a representative example for elucidating the step-by-step process and predicted results of PSEs using the novel RV negative strategy. Similarly, PSEs of the other 16 representative strains of pathogenic *L. interrogans* were predicted following same strategy as *str*.56601. First of all, PSORTb3.0, CELLO, and SOSUI-GramN were used to predict subcellular localization of these proteins by majority voting strategy. Proteins predicted as CYTs and IMPs by at least two of the three bioinformatic tools were defined as consensus CYTs and IMPs and were directly removed from further study. Proteins predicted as CYTs or IMPs by only one of the three tools were labeled as non-consensus CYTs or IMPs, respectively. The remaining proteins were labeled as PSEs. Then, both the non-consensus CYTs with no signal peptides predicted by all of SignalP3.0, TatP and SecretomeP and the non-consensus IMPs with positive transmembrane structures predicted by TMHMM or Phobius were defined as Non-PSEs and removed from further study, whereas the remaining non-consensus CYTs with positive signal peptide and non-consensus IMPs with no transmembrane structure were added into the predicted PSEs. In addition, SignalP3.0, Tat and SecretomeP as well as BOMP, TMBETADISC-RBF, and LipoP were utilized to further investigate extracellular features of these PSEs. Finally, pan-genome analysis of the predicted PSEs among the 17 pathogenic *L. interrogans* strains identified the core, dispensable, and unique PSEs. And the core PSEs with high antigenicity values predicted by the VaxiJen server were determined as final vaccine antigen candidates. PSE, potential surface-exposed proteins; CVPSE, Conserved Vaxijen antigenicity predicted PSE.

A novel RV approach employing a negative selection strategy was used in this work (Figure 1). At first, the three currently used bioinformatic tools, PSORTb3.0 (Yu et al., 2010), CELLO (Yu et al., 2004), and SOSUI-GramN (Imai et al., 2008), were used to predict subcellular localization of these proteins by a majority voting strategy. Proteins predicted as CYTs by at least two of the three bioinformatic tools were defined as consensus CYTs. Similarly, proteins predicted as IMPs by at least two of the three tools were defined as consensus IMPs. Proteins predicted as CYTs or IMPs by only one of the three tools were labeled as non-consensus CYTs or IMPs, respectively. The remaining proteins were labeled as PSEs. Thus, the predicted results were preliminarily divided into three groups: consensus CYTs/IMPs, non-consensus CYTs/IMPs, and PSEs. The consensus CYTs and IMPs as non-PSEs were directly removed from further study. Non-consensus CYTs and IMPs were further analyzed by combination of additional bioinformatic tools. If these non-consensus CYTs were predicted to be negative by SignalP3.0 (Bendtsen et al., 2004b), TatP (Juncker et al., 2003), and SecretomeP (Bendtsen et al., 2004a), they were removed from further analysis. Non-consensus CYTs with positive signal peptide results were retained as PSEs. Non-consensus IMPs with transmembrane structures predicted by TMHMM (Krogh et al., 2001) or Phobius (Kall et al., 2004) were also removed for further study. Non-consensus IMPs with no transmembrane structures predicted by TMHMM and Phobius were retained as PSEs. Thus, the remaining proteins classified as PSEs were categorized as follows: (1) ECPs or periplasmic proteins predicted by SignalP3.0, Tat and SecretomeP; (2) OMPs predicted by BOMP (Berven et al., 2004), TMBETADISC-RBF (Ou et al., 2008) and LipoP (Juncker et al., 2003); and (3) proteins with unknown localization. Finally, based on amino acid sequences, the antigenicity value of each PSE was predicted using the VaxiJen server with default parameter “bacteria” and the threshold of 0.5 (Doytchinova and Flower, 2007).

### Bioinformatic tools used in reverse vaccinology

Subcellular localization of *L. interrogans* proteins was predicted by PSORTb, CELLO and SOSUI-GramN. These were classified into CYTs, IMPs, periplasmic proteins (PMs), OMPs, or ECPs. SignalP3.0, TatP, SecretomeP, LipoP, TMBETADISC-RBF, and BOMP were used for further extracellular feature prediction. String database was used for analyzing protein–protein interactions (PPI) of *L. interrogans* PSEs (Franceschini et al., 2013).

### Pangenomic analysis of predicted PSEs among 17 leptospiral strains

Reciprocal blast with bidirectional best hit (BBH) and *e*-values of 10^−10^ were used for ortholog clustering of *L. interrogans* in a pan-genome analysis. Additionally, in order to avoid homologous mismatches, both the coverage and identity percent of cut-offs were set to at least 50%. The concepts of core, dispensable, and unique PSEs were used in this study according to the pan-genome classification. Core PSEs were highly conserved among all 17 strains. Dispensable PSEs and unique PSEs existed in less than 16 strains and exclusively in only one strain, respectively. Finally, these core PSEs with high antigenicity values predicted by the VaxiJen server were determined as the final vaccine antigens candidates against leptospirosis.

## Results

### General information of selected *L. interrogans* strains

A total of 17 leptospiral strains covering 11 different serovars and 17 STs were selected for analysis (Table 1). Among these strains, serovars Bataviae, Grippotyphosa, and Pyrogenes consisted of three different STs. The present study was focused mainly on those selected strains that are the most common serovars in China; further, the STs associated with evolutionary information were taken into account (Varni et al., 2013).

### Prediction schema of PSEs by the negative selection method

**Figure 2:**
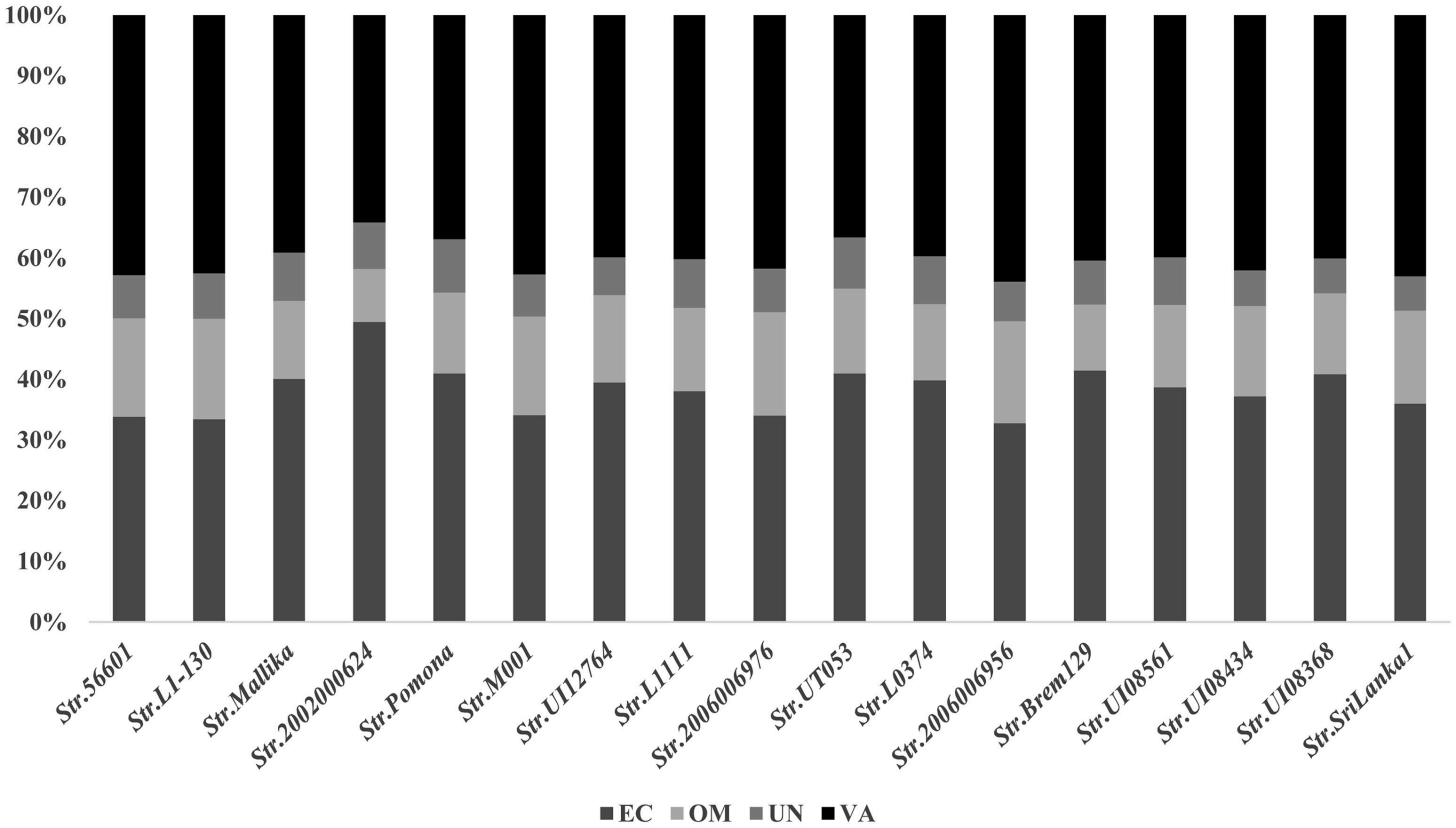
**Subcellular localizations of these PSEs among the 17 representative strains of Pathogenic *L. interrogans***. EC, extracellular; OM, outer membrane; UN, unknown; VA, variable (proteins with multiple locations-EC or OM).

The new combined RV strategy is illustrated by Figure 1. We chose *L. interrogans str*.56601 as an example. A total of 3,702 proteins were analyzed using our novel RV strategy; 2,706 consensus CYTs and IMPs, 666 non-consensus proteins, and 330 PSEs were predicted. Among these 2,706 proteins, 2,166 proteins were predicted as CYTs and 540 as IMPs by at least two of the three software (PSORTb3.0, CELLO and SOSUI-GramN). Moreover, these 666 non-consensus proteins predicted as CYT or IMP by only one of the three software were further assessed according to the following rules: For example, LA_0012 was predicted to be unknown in PSORT, OMP in Cello and CYT in SoSui-GramN, respectively; And LA_0009 was predicted to be unknown in PSORT, OMP in Cello, IMP in SoSui-GramN. A total of 398 non-consensus proteins like LA_0012 and 157 proteins like LA_0009 were subdivided as non-consensus CYTs and non-consensus IMPs, respectively. In addition, the remaining 111 non-consensus proteins like LA_0293 with unknown location in PSORTb, CYT in Cello and IMPs in SoSui-GramN, were defined as both non-consensus CYTs and IMPs. Therefore, the 666 non-consensus proteins were divided into 509 non-consensus CYTs (398 plus 111) and 268 non-consensus IMPs (157 plus 111). Among the 509 non-consensus CYTs, 311 were predicted negative using the three programs (SignalP3.0, TatP, and SecretomeP) and were removed from further analysis. There were 198 non-consensus CYTs with positive signal peptide results; these were retained as PSEs. Another 268 non-consensus IMPs were further analyzed by TMHMM (Krogh et al., 2001) or Phobius (Kall et al., 2004). One hundred and twenty-seven of these were predicted to have transmembrane structures and eliminated from further study. The remaining 141 with no transmembrane structure were retained and classified as PSEs. Finally, 303 were also predicted to be PSEs out of the 666 non-consensus proteins. Altogether, in addition to the 330 PSEs mentioned above, we predicted a total of 633 PSEs from 3,702 proteins in this study. Among them, the subcellular localization of 45 proteins was unknown and the remaining proteins were almost all predicted as OMPs or ECPs. The predicted PSEs were as high as 92.8% (588/633). The detailed information of PSEs in the remaining strains identified was shown in Figure 2.

### Pan-genome analysis of predicted PSEs among 17 leptospiral strains

**Figure 3:**
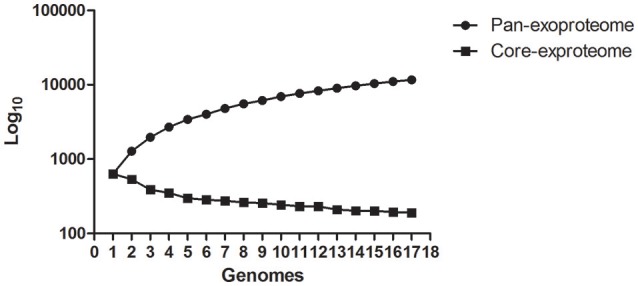
**Calculation of core- and pan-genome sizes of Pathogenic *L. interrogans* including exponential law models**.

**Figure 4:**
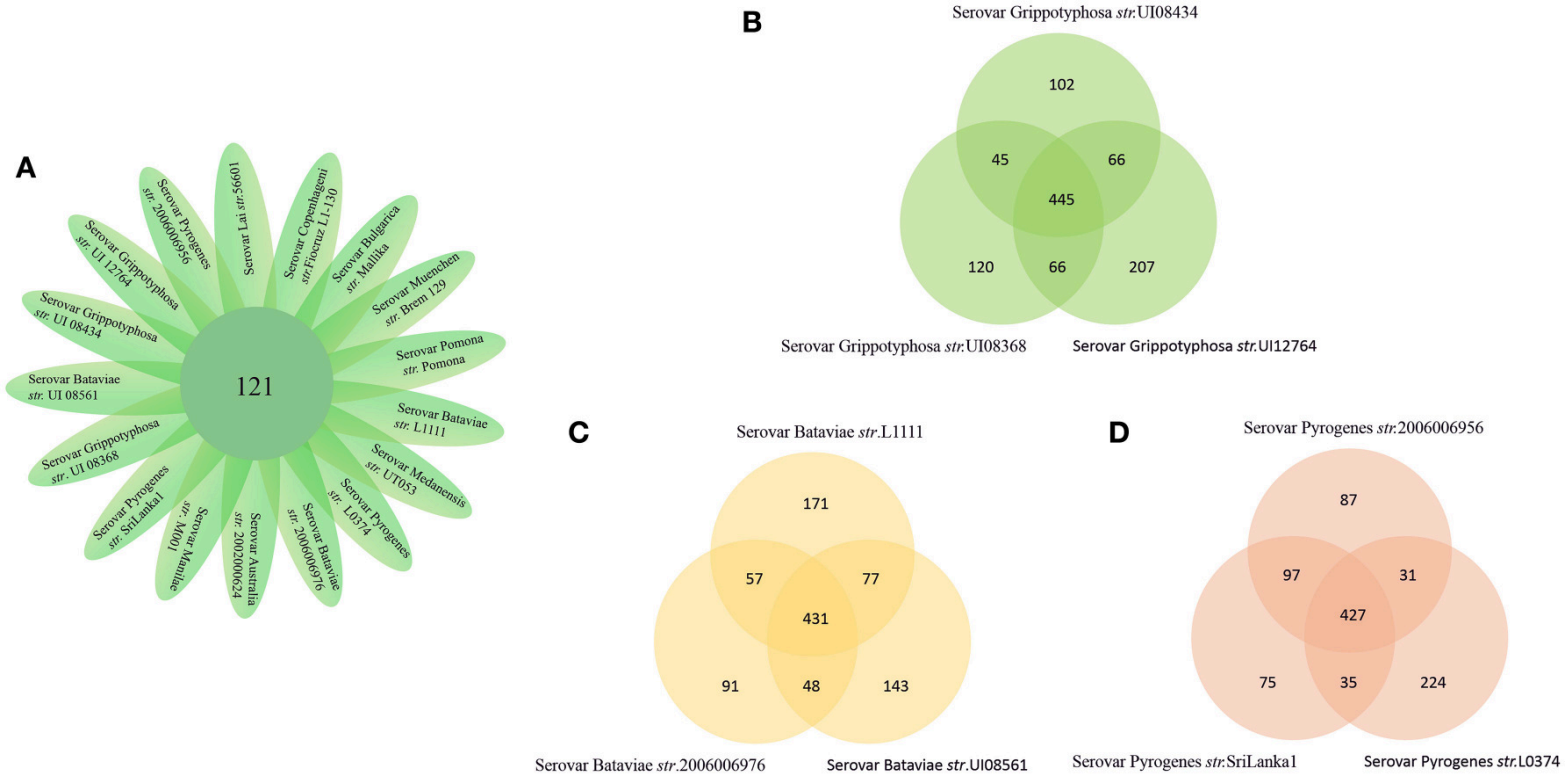
**Pan-genome representation for the 17 representative strains of Pathogenic *L. interrogans*. (A)** Core genes of the 17 *L.interrogans* strains. In our *L.interrogans* candidates, nine strains belong to three different serovars **(B–D)**. Show pan-genome of the three serovars themselves.

The number of predicted PSEs in the various strains of *L. interrogans* ranged from 600 to 780 (Figure 2). Gene accumulation curves showed that core genome size fits an exponential decay curve that reached a plateau at 11,043 proteins, whereas the pan PSE grouping fits a power law curve, suggesting the 17 leptospiral strains selected are sufficient to characterize pan core PSEs (Figure 3). Among the 1,103 leptospiral ortholog clusters, 190 core PSEs (17.2%) and 913 dispensable PSEs (82.8%) were shared by all 17 of *L. interrogans* strains and partly conserved among 2–16 strains, respectively. Furthermore, the pan PSEs included 861 unique PSEs that were found only in one strain. The numbers of unique PSEs in each strain range from 17 (serovar Manilae *str*.M001) to 103 (serovar Medanensis *str*.UT053). The dispensable and unique PSEs might be related to different serotypes. The detailed information of all strains and those three dependent serovars was shown in Figure 4. In the present study, our main goal was to predict potential novel protective antigens for the development of universal vaccines against leptospirosis; special attention was given to the 121 high antigenic PSEs from 190 core PSEs, including 37 ECPs, 83 OMPs, and 1 unknown protein localization (see Table 2). As more than 40% of *L. interrogans* proteins have been annotated as hypothetical proteins, further study of these proteins' functions is needed. Among them, only 55 were categorized into the following COG groups, including main cell wall/membrane/envelope biogenesis (9); function unknown (9); cell motility (7); inorganic ion transport and metabolism (5); general function prediction only (4); Posttranslational modification, protein turnover, chaperones (4); Carbohydrate transport and metabolism (3); Energy production and conversion (2), etc. 16 PSEs were predicted as being involved in(a) cell wall/membrane/envelope biogenesis or (b) cell motility, which are related to the classical function of PSEs (Table S1). In addition, we predicted dispensable and unique PSEs in our pan-genome analysis. For instance, there were 28 unique PSEs in *str*.56601 and 27 in *str*. Fiocruz L1-130 (Table S2).

**Table 2:** **The detailed information of the final 121 PSEs with predicted high antigenicity among the 17 representative strains of pathogenic *L.interrogans***.

***Str***. **56601 Locus**	***Str***. **L1-130 Locus**	**Osmolarity *in vivo*/*in vitro*) (Matsunaga et al., 2007)**	**Low iron (Lo et al., 2010)**	**Serum (Patarakul et al., 2010)**	**Temperature P-E (Lo et al., 2006)**	**Temperature upshift (Lo et al., 2006)**	**Lai/IPAV (Zhong et al., 2011)**	**COG**	**Product**	**Localization**	**Vaxijen antigenicity**
LA_0022	LIC10021	–	–	–	–	–	–	–	Hypothetical protein	OMPs	0.7022
LA_0071	LIC10064	–	–	–	–	–	–	COG4731S	Hypothetical protein	ECPs	0.7164
LA_0074	LIC10067	–	–	–	–	–	–	–	Hypothetical protein	ECPs	0.6286
LA_0075	LIC10068	–	–	↓	–	–	–	–	Hypothetical protein	ECPs	0.5617
LA_0136	LIC10123	–	–	–	–	–	↓	COG4254S	Hypothetical protein	OMPs	0.6461
LA_0303	LIC10260	–	–	–	–	–	–	–	Hypothetical protein	OMPs	0.6756
LA_0322	LIC10280	–	–	–	–	–	–	–	fibronectin binding protein	OMPs	0.7026
LA_0333	LIC10288	–	–	–	–	–	–	COG0603R	PP-loop superfamily ATPase	OMPs	0.6805
LA_0346	LIC10298	–	–	–	–	–	–	COG1558N	Flagellar basal body rod protein FlgC	ECPs	0.633
LA_0357	LIC10307	–	–	–	–	–	–	COG0412Q	Dienelactone hydrolase family protein	OMPs	0.6134
LA_0364	LIC10313	–	–	–	–	–	–	–	Hypothetical protein	OMPs	1.094
LA_0410	LIC10359	–	–	–	–	–	–	COG2834M	Outer membrane lipoprotein-sorting protein	OMPs	0.6661
LA_0419	LIC10368	–	–	–	–	–	–	–	Hypothetical protein	OMPs	0.7143
LA_0430	LIC10377	–	–	–	–	↑	–	–	Hypothetical protein	OMPs	0.8101
LA_0505	LIC13050^*^	–	–	–	–	–	↓	–	Hypothetical protein	OMPs	0.8807
LA_0568	LIC13002	–	–	–	–	↓	–	COG2067I	Fatty acid transport protein	OMPs	0.6682
LA_0589	LIC12986	–	↑	–	–	–	–	–	hypothetical protein	OMPs	0.5822
LA_0591	LIC12985	–	–	–	↑	↑	–	–	Hypothetical protein	ECPs	0.5631
LA_0663	LIC12930	–	–	↑	–	–	–	–	Hypothetical protein	ECPs	0.5964
LA_0862	LIC12765	–	–	↓	–	↓	↓	COG2077O	Thiol peroxidase	ECPs	0.5274
LA_1122	LIC12558	–	–	–	↑	↑	–	–	Hypothetical protein	OMPs	0.7834
LA_1159	LIC12525	–	–	–	–	–	–	–	Hypothetical protein	OMPs	0.5873
LA_1167	LIC12519	–	–	–	–	–	–	–	Hypothetical protein	OMPs	0.765
LA_1168	LIC12518	–	–	–	–	–	–	–	Hypothetical protein	ECPs	0.6311
LA_1180	LIC12509	–	–	–	↑	–	–	–	Hypothetical protein	ECPs	0.6433
LA_1192	LIC12499	–	–	–	↑	↑	–	–	Hypothetical protein	OMPs	0.564
LA_1356	LIC12374	–	–	–	–	–	–	COG4206H	TonB-dependent outer membrane receptor	OMPs	0.6274
LA_1404	LIC12337	–	–	–	–	–	↑	–	hypothetical protein	OMPs	0.5464
LA_1458	LIC12295	–	–	–	–	–	–	COG1134GM	ABC transporter ATP-binding protein	OMPs	0.5095
LA_1499	LIC12259	–	–	–	–	–	–	COG0026F	Phosphoribosylaminoimidazole carboxylase ATPase subunit	OMPs	0.6708
LA_1507	LIC12254	–	–	–	–	–	–	COG4775M	Hypothetical protein	OMPs	0.5181
LA_1508	LIC12253	–	–	–	↑	↑	↓	COG4775M	Hypothetical protein	ECPs	0.599
LA_1897	LIC12002	–	–	↓	–	–	–	COG0753P	Catalase	ECPs	0.5618
LA_1931	LIC11975	–	–	–	–	–	–	–	Hypothetical protein	OMPs	0.802
LA_1968	LIC11935	–	–	–	–	–	↓	–	Hypothetical protein	OMPs	0.5621
LA_2069	LIC11846	–	–	–	–	–	–	–	Hypothetical protein	OMPs	1.0393
LA_2105	LIC11813	–	–	–	–	↑	–	COG1886NU	Flagellar motor switch protein	OMPs	0.5388
LA_2186	LIC11739	–	–	–	–	–	–	COG0405E	gamma-glutamyltranspeptidase	OMPs	0.5669
LA_2272	LIC11665	–	–	–	–	–	–	–	Hypothetical protein	OMPs	0.6501
LA_2316	LIC11625	–	–	–	–	–	–	COG2013S	Hypothetical protein	ECPs	0.8212
LA_2377	LIC11568	–	–	–	–	–	–	COG2133G	Glucose/sorbosone dehydrogenase	OMPs	0.6489
LA_2498	LIC11467	–	–	–	–	–	–	COG0739M	M23 family metalloendopeptidase	OMPs	0.6045
LA_2538	LIC11435	–	–	–	–	–	–	COG5184DZ	Regulator of chromosome condensation	OMPs	0.6316
LA_2550	LIC11424	–	–	–	–	–	↑	COG0596R	Esterase/lipase	ECPs	0.7281
LA_2595	LIC11388	–	–	–	–	–	–	–	Hypothetical protein	OMPs	0.863
LA_2601	LIC11382	–	–	–	–	↑	–	–	Hypothetical protein	OMPs	0.5082
LA_2613	LIC11370	–	–	–	–	–	–	COG0545O	FKBP-type peptidylprolyl isomerase	OMPs	0.6189
LA_2617	LIC11366	–	–	–	–	–	–	–	Hypothetical protein	OMPs	0.7008
LA_2641	LIC11345	–	–	–	–	–	–	COG1886NU	Endoflagellar motor switch protein	OMPs	0.6637
LA_2672	LIC11320	–	–	–	–	–	–	–	Hypothetical protein	OMPs	0.5604
LA_2741	LIC11271^*^	–	–	–	–	–	–	–	Hypothetical protein	ECPs	0.715
LA_2742	LIC11270	–	–	–	–	–	–	COG1629P	Ferrichrome-iron receptor	ECPs	0.5877
LA_2746	LIC11268	–	–	–	–	–	–	–	Hypothetical protein	OMPs	0.7177
LA_2757	LIC11259	–	–	–	–	↓	–	–	Hypothetical protein	OMPs	0.517
LA_2764	LIC11254	–	–	–	–	↑	–	COG1613P	Sulfate ABC transporter substrate-binding protein	OMPs	0.5849
LA_2796	LIC11228	–	–	↓	–	–	–	COG0768M	Transpeptidase/penicillin binding protein	OMPs	0.6239
LA_2815	LIC11213	–	–	–	–	–	–	COG1792M	Rod shape-determining protein MreC	OMPs	0.6909
LA_2823	LIC11207	–	–	–	↑	↑	–	–	Hypothetical protein	ECPs	0.7306
LA_2848	LIC11188	–	–	–	–	↓	–	–	Hypothetical protein	OMPs	0.7231
LA_2849	LIC11187	–	–	–	–	↓	–	–	Hypothetical protein	ECPs	0.5677
LA_2850	LIC11186	–	–	–	–	–	–	–	Hypothetical protein	ECPs	0.6522
LA_2854	LIC11184	–	–	↓	–	–	–	COG1749N	Flagellar hook protein FlgE	OMPs	0.6733
LA_2949	LIC11112	–	–	–	↓	↓	–	COG1843N	Flagellar hook assembly scaffolding protein	OMPs	0.5626
LA_2958	LIC11103	–	–	–	↑	↑	–	COG3144N	Flagellar protein	OMPs	0.5731
LA_2975	LIC11087	–	–	–	–	–	–	COG0265O	Serine protease	OMPs	0.5597
LA_2992	LIC11074	–	–	–	–	–	–	COG2267I	Hypothetical protein	ECPs	0.7857
LA_2993	LIC11073	–	–	–	–	–	–	COG1858P	Cytochrome c peroxidase	OMPs	0.6704
LA_2998	LIC11067	–	–	–	–	–	–	–	Hypothetical protein	ECPs	0.8504
LA_3026	LIC11052^*^	–	–	–	–	–	–	–	Hypothetical protein	OMPs	0.6387
LA_3050	LIC11040	–	–	–	–	↑	–	–	Hypothetical protein	OMPs	0.5837
LA_3064	LIC11030	–	–	–	–	–	–	COG1664M	Cell shape determination protein	OMPs	0.7079
LA_3097	LIC11003	–	–	–	–	–	–	–	Hypothetical protein	OMPs	0.7837
LA_3138	LIC10973	–	–	–	–	–	–	–	Hypothetical protein	ECPs	0.6481
LA_3145	LIC10968	↑	–	–	–	–	–	–	Hypothetical protein	OMPs	0.6892
LA_3150	LIC10963	–	–	–	–	–	–	COG1652S	Hypothetical protein	ECPs	0.6863
LA_3210	LIC10920	–	–	–	–	↓	–	–	OmpL1	OMPs	0.9344
LA_3268	LIC10873	–	–	–	–	–	–	–	Hypothetical protein	ECPs	0.5264
LA_3303	LIC10845	–	–	–	–	–	–	–	Hypothetical protein	ECPs	0.6679
LA_3319	LIC10833	–	–	–	–	–	–	COG2010C	Hypothetical protein	ECPs	0.6143
LA_3410	LIC10760	–	–	–	–	↑	–	–	Hypothetical protein	ECPs	0.9564
LA_3454	LIC10723	–	–	–	–	–	–	COG3865S	Hypothetical protein	OMPs	0.5181
LA_3468	LIC10714	–	–	–	–	–	–	–	Hypothetical protein	OMPs	0.5529
LA_3469	LIC10713	–	–	–	–	–	–	COG2353S	Hypothetical protein	OMPs	0.6583
LA_3470	LIC10712	–	–	–	–	–	–	COG1345N	Flagellar hook-associated protein FliD	ECPs	0.5272
LA_3501	LIC10686	–	–	–	–	–	–	COG3487P	Hypothetical protein	OMPs	0.57
LA_3508	LIC10683	–	–	–	–	–	–	COG3489R	Hypothetical protein	ECPs	0.7949
LA_3571	LIC10628	–	–	–	–	–	–	COG3794C	Methylamine utilization protein	OMPs	0.6392
LA_3711	LIC10520	–	–	–	–	–	–	–	Hypothetical protein	ECPs	0.7514
LA_3778	LIC10464	↑	–	↑	–	↑	–	COG2885M	OmpA family protein	OMPs	0.5249
LA_3834	LIC13066	↑	–	–	–	–	–	COG3607R	Glyoxalase/bleomycin resistance protein/dioxygenase	OMPs	0.6016
LA_3838	LIC13070	–	–	–	–	–	–	–	Hypothetical protein	OMPs	0.5302
LA_3849	LIC13076	–	–	–	–	–	–	–	Hypothetical protein	OMPs	0.6748
LA_3853	LIC13078	–	–	–	–	–	–	–	Hypothetical protein	OMPs	0.5234
LA_3867	LIC13086	↑	↑	–	–	↑	–	–	Hypothetical protein	OMPs	0.5336
LA_3870	LIC13089	–	–	–	–	–	–	–	Hypothetical protein	OMPs	0.5439
LA_3881	LIC13101	–	–	–	–	↑	–	–	Hypothetical protein	OMPs	0.7749
LA_4059	LIC13238	–	–	–	–	–	–	–	Hypothetical protein	OMPs	0.7565
LA_4083	LIC13255	–	–	–	↓	↓	–	COG2303E	Cholesterol oxidase precursor	OMPs	0.667
LA_4144	LIC13306	–	–	–	–	–	–	–	Hypothetical protein	ECPs	0.5584
LA_4178	LIC13334	–	–	–	–	–	–	–	Hypothetical protein	ECPs	0.5203
LA_4202	LIC13354	–	–	–	↓	↓	–	COG4642S	Hypothetical protein	OMPs	0.6064
LA_4203	LIC13355	–	–	–	–	–	↓	COG0331I	Fatty acid synthase subunit beta	OMPs	0.5784
LA_4272	LIC13418	–	–	–	–	–	↓	–	Hypothetical protein	OMPs	0.7046
LA_4291	LIC13434	–	–	–	–	–	–	–	Hypothetical protein	OMPs	0.5418
LA_4293	LIC13436	–	–	–	–	–	↓	–	polysaccharide deacetylase	OMPs	0.6614
LA_4335	LIC13477	–	–	–	–	–	–	–	Hypothetical protein	OMPs	0.6873
LB_001	LIC20001	–	–	–	–	–	↓	–	Hypothetical protein	OMPs	0.6864
LB_056	LIC20042^*^	–	–	–	–	–	–	–	Hypothetical protein	ECPs	0.8468
LB_072	LIC20056	–	–	–	–	–	–	–	Hypothetical protein	ECPs	0.5987
LB_098	LIC20077	–	–	–	–	–	–	–	Hypothetical Protein	ECPs	0.5732
LB_110	LIC20087^*^	–	–	–	–	–	–	COG5010U	TPR repeat-containing protein	OMPs	0.5788
LB_191	LIC20151	–	–	–	–	–	–	–	Hypothetical protein	OMPs	0.7302
LB_192	LIC20152	–	–	–	–	–	–	COG1022I	Long-chain-fatty-acid–CoA ligase	OMPs	0.6367
LB_194	LIC20153	–	–	–	–	–	-	COG0726G	Xylanase/chitin deacetilase	OMPs	0.6861
LB_242	LIC20185	–	–	↓	–	–	–	–	Putative outermembrane protein	OMPs	0.5456
LB_258	LIC20197	–	–	–	–	–	–	COG4206H	Putative TonB-dependent outer membrane receptor protein	OMPs	0.5271
LB_268	LIC20205	–	–	–	–	–	↓	–	Hypothetical protein	ECPs	0.5724
LB_277	LIC20212	–	–	–	–	↓	–	COG4254S	Hypothetical protein	ECPs	0.6918
LB_279	LIC20214	–	–	–	–	–	↑	COG4870O	Cysteine protease	OMPs	0.6034
LB_280	LIC20215	–	–	–	–	–	–	COG2267I	Hypothetical protein	ECPs	0.5028
LA_1462	LIC12293	–	–	–	–	–	↑	COG1633S	Hypothetical protein	Unknown	0.5146

* *Proteins were confirmed to be reactive with human serum (Lessa-Aquino et al., 2013). Osmolarity (in vivo/in vitro), physiologic osmolarity compared with those at low osmolarity; Low iron, genes differentially expressed under low-iron conditions; Serum, gene changed in serum; Temperature P-E, genes changes in physiological vs. environmental temperatures; Temperature upshift, genes changes in overnight 37° upshift vs. 30° long term; “↓,” down –regulated; “↑,” up-regulated; “–”, unknown or without changes*.

## Discussion

PSEs of pathogens are potential immune targets for the host immune system (Solis and Cordwell, 2011). In this study, we analyzed the PSEs of 17 leptospiral representative strains covering 11 main serovars and 17 STs, and identified potential vaccine candidates or virulence factors.

**Figure 5:**
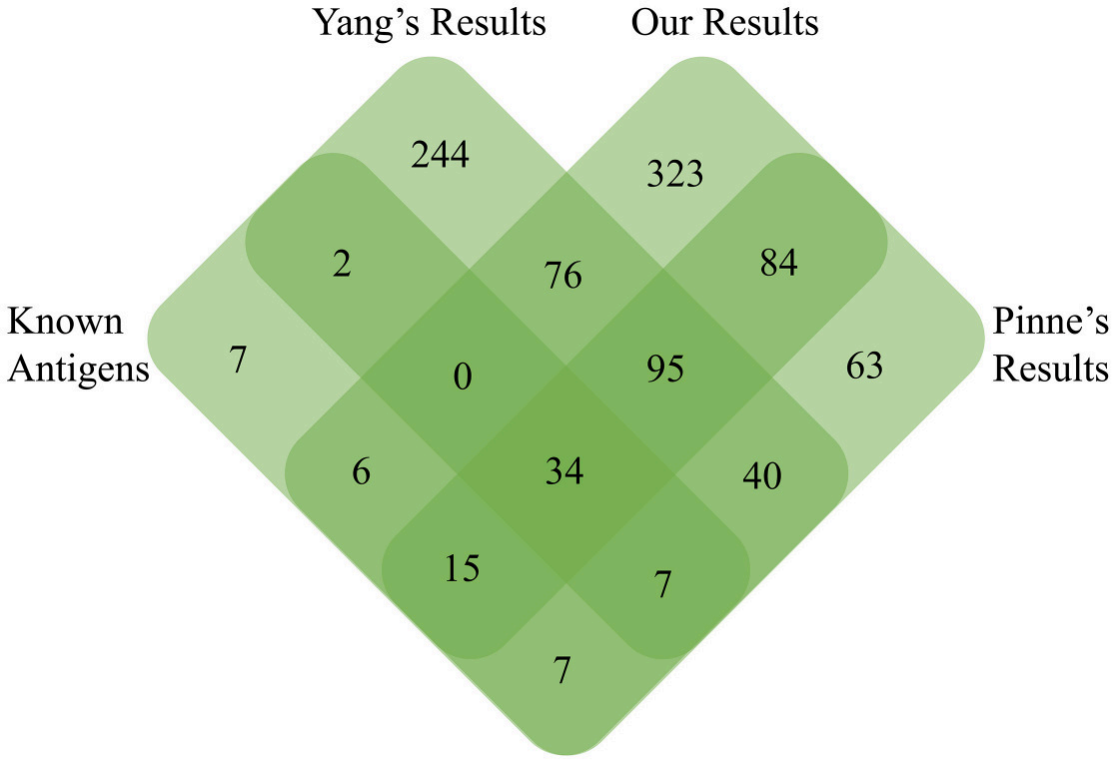
**Venn diagram detailed the unique and common PSEs among our negative-screening, Yang's positive-screening, and Pinne's OMP array results with known experimentally identified surface-exposed antigens**.

Recently, we identified a total of 33 highly reliable ECPs in serovar Lai *str*.56601 using a newly modified protein-free medium, and 26 of them were found in predicted PSEs of *str*.56601 in the current study, including LipL32, LipL36, LipL48, LenC, LenE, TonB receptor, OmpA family protein, and 8 putative lipoproteins and 6 hypothetical proteins (Zeng et al., 2013). In addition, a novel *L. interrogans* OMP microarray was developed and contained a total of 366 predicted lipoproteins and transmembrane OMPs (Pinne et al., 2012). About 70% (239/346) of these OMPs or lipoproteins in the protein array were found in our predicted PSEs of *str*. Fiocruz L1-130. It has been reported that 1,026 proteins in the TX-114 OMP-enriched fraction were found from the transcriptional and translational responses to temperature shift by high-throughput liquid chromatography tandem mass spectrometry (LC/MS-MS); however, only 154 of the 1026 proteins were found in our predicted PSEs of *str*.56601. The significant discrepancies could be due to lower coverage of OMPs or lipoproteins within the 1,026 proteins, which comprised no more than 80 predicted or known OMPs or lipoproteins (Lo et al., 2009). In order to comprehensively evaluate the advantages and disadvantages of our negative-screening RV strategy, we further compared another three different data sets including experimentally identified 78 surface-exposed antigens or virulence factors (see Table S3). 499 PSEs of *L. interrogans* were identified by a positive-selection RV strategy as previously described by Yang et al. (2006) and 346 OMPs/lipoproteins of *L. interrogans* in the *L. interrogans* OMP array (Pinne et al., 2012), with our negative-screening results (See Figure 5 and Table S3). Among all 78 known surface-exposed antigens, 63, 55, and 43 were identified in the OMP array (Pinne et al., 2012), in this study and Yang's studies (Yang et al., 2006), respectively. Actually, the highest consistency between protein array result and the known surface-exposed antigens might mainly be due to more than 90% (70/78) of known antigens being located in the outer membrane. Moreover, there are 95 common OMPs/Lipoproteins among Yang's, Pinne's and our study's antigen inventory. There were 84 common OMPs/Lipoproteins between Pinne's and our study while there were only 40 proteins between Pinne's and Yang's study. Thus, for OMP/Lipoprotein, our negative RV strategy predicted more proteins than that of Yang's positive RV strategy. However, the information of extracellular proteins in pathogenic *Leptospira* spp. is still limited. Further, studies to identify more ECPs and to assess the prediction precision of the two different RV strategies are needed.

In this study, pan-genome analysis showed 121 highly antigenic PSEs conserved completely among all 17 strains. Except for several known proteins, including LipL45, OmpL1, and LigB, the majority of these candidates are identified in *Leptospira* for the first time (Pinne et al., 2012). Among the 121 PSEs, the most promising new vaccine antigens appear to be hypothetical proteins (LA_2741), BatC (LB_056), and lipL71/LruA (LA_3097). LA_2741 and BatC were recognized in leptospirosis patients and identified as differentially reactive antigens between acute- or convalescent-phase leptospirosis patients and healthy individuals (Lessa-Aquino et al., 2013). The lipoprotein LruA, present in pathogenic *L. interrogans* but not in non-pathogenic *L. biflexa*, could induce high levels of humoral antibody responses in equine uveitis eyes and in sera of humans with leptospiral uveitis (Verma et al., 2005). Thus, these three PSEs could be worthy of further investigation as novel vaccine candidates and/or diagnostic markers for leptospirosis because of common features, including surface-exposed localization, universal conservation, and eliciting strong antibody production in patients (Verma et al., 2005).

**Figure 6:**
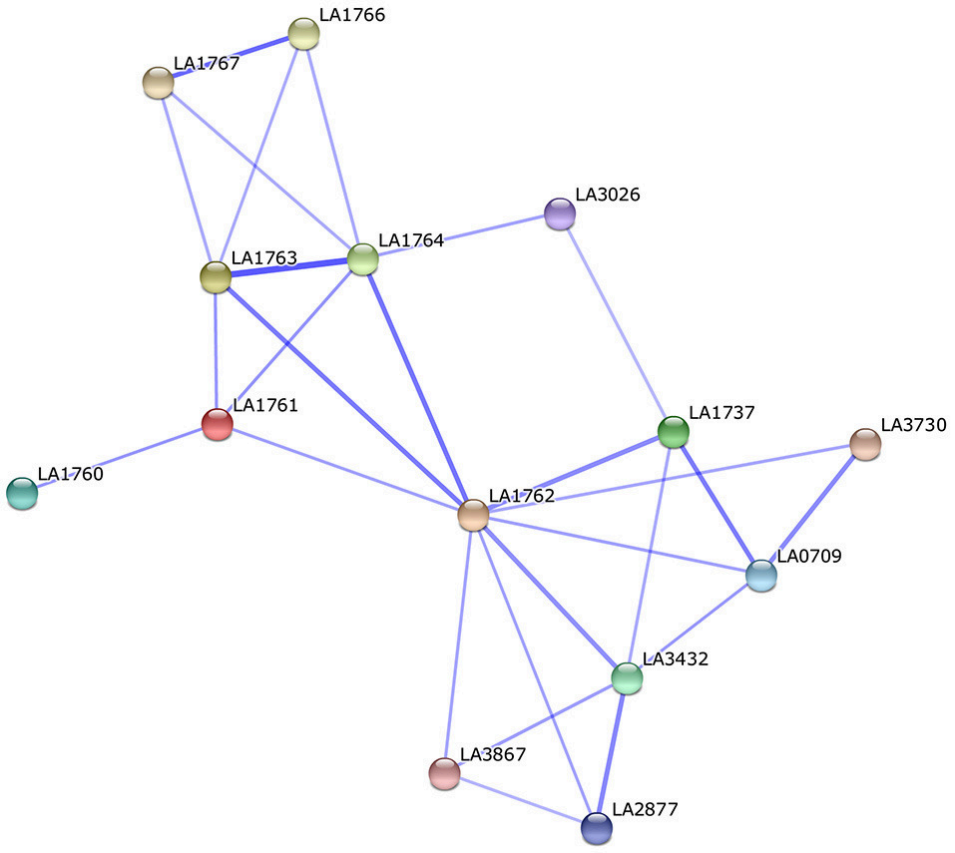
**Protein-protein interaction of the potential virulence factors (LA_1761 to LA_1764) located in the 54 kb circular prophage of *str*.56601**.

Surface-exposed proteins generally comprise a wide array of virulence factors involved in pathogen–host interactions and are responsible for causing disease. Comparing our predicted results to the previous leptospiral OMP microarray data (Pinne et al., 2012), 11 of 15 fibronectin-binding proteins were found in the predicted PSEs of *str*. Fiocruz L1-130, which were subdivided into four core PSEs (hypothetical protein, TonB-dependent receptor, iron-regulated lipoprotein, and OmpA family proteins) and seven dispensable PSEs (lipoprotein, Lsa66, leucine-rich repeat protein, sphingomyelinases 2 and 3; Pinne et al., 2012). All four core PSEs are involved in adherence to fibronectin during the initial attachment stage of infection and have significant potential to exhibit key roles in the pathogenesis of leptospirosis. For example, TonB-dependent receptor (LA_3468), and iron-regulated lipoprotein (LA_3469) are related to iron uptake, which is essential for pathogenic leptospires (Murray et al., 2008). In our study, iron-regulated lipoprotein (LA_3469) was confirmed to be up-regulated at 37°C as compared to 28°C and could activate the host's immune system to produce a high-level antibody response (our unpublished data), indicating this protein might have an indispensable function in the pathogenesis of *L. interrogans*. The dispensable PSEs sphingomyelinases Sph2 and Sph3 (LA_1029 and LA_4004) showed distinctly different conservation. It has been confirmed that Sph2 secreted as sphingomyelinase hemolysin has strong hemolytic activity against sheep erythrocytes as well as cytotoxic activity against mouse lymphocytes and macrophages (Zhang et al., 2005, 2008). Thus, Sph2 might be important as a novel virulence factor involved in leptospiral pathogenesis and might be associated with virulence differences among different leptospirosis serovars. Another dispensable PSE is the leucine-rich repeat protein (LA_3028) found exclusively in the highly pathogenic strains: *str*.56601 and *str*. Fiocruz L1-130. The leucine-rich repeat protein (LRR) has been reported frequently as a virulence factor in numerous pathogens involved in cell adhesion, invasion, and stimulation of host defense mechanisms (Kobe and Kajava, 2001; Brinster et al., 2007). The leucine-rich repeat protein was identified as a fibronectin-binding protein and it should be, at least partly, related to the high virulence of *str*.56601 and *str*. Fiocruz L1-130. The other core PSE like hypothetical protein LA_0505 predicted as a secretion protein through non-classical pathway, has been shown to bind some host extracellular matrices (such as laminin, plasma fibronectin, fibrinogen, etc.) and play an important role in adhesion of *L. interrogans* (Pinne et al., 2012). Interestingly, LA_0505 was found in the supernatant of *L. interrogansstr*. 56601 and up-regulated *in vivo* in our recent study (Zeng et al., 2013). Moreover, LA_0505 has a BIG domain as Ca^2+^-binding modules during the process of leptospirosis (Raman et al., 2010). The potential virulence factors in predicted PSEs are the four hypothetical proteins LA_1761–1764 identified here. These four PSEs are located in the 54 kb separate circular prophage of *str*.56601, which was inserted into the larger chromosome at the same time; however, the 54 kb prophage was absent from the genome of *str*. Fiocruz L1-130 (Bourhy et al., 2007). Until now, there was no experimental evidence suggesting these four proteins might be associated with the virulence of *Leptospira*; however, PPI analysis in the string database suggested that the four proteins interact mostly with other hypothetical proteins in the PPI network (Figure 6). LA_1762 interacts with lipoproteins LA_3730 and LA_3867, both of which were identified as putative extracellular proteins and thus were recommended as novel candidates for the development of leptospirosis vaccines (Viratyosin et al., 2008). LA_3867 was identified as one of the most strongly up-regulated genes of pathogenic *L. interrogans* at physiologic osmolarity as compared to low osmolarity, indicating over-expression of LA_3867 in pathogenic leptospires might be associated with transition from survival in the outside environment to infection of mammalian hosts (Matsunaga et al., 2007). Therefore, as an interacting partner of LA_3867, LA_1762 could have a crucial role in successful establishment of host infection.

## Conclusions

A new RV negative-screening strategy combined with pan-PSE analysis was used to screen PSEs among 17 *L. interrogans* strains. We identified 190 core PSEs, 913 dispensable PSEs, and 861 unique PSEs. Further, antigenicity analysis finally identified 121 highly antigenic PSEs as potential vaccine candidates from the 190 core PSEs, which include several known antigens, including LipL45, OmpL1, and LigB, and the vast majority of newly identified potential vaccine candidates against leptospirosis. At the same time, we also characterized many potential virulence factors in our inventory of predicted PSEs. Our prediction results may accelerate vaccine development against leptospirosis and deepen our understanding of leptospiral virulence mechanisms. Moreover, this *in silico* strategy combined with pan-genome analysis could be a routine method of reverse vaccinology applied widely to similar pathogens. Further, cloning, expression, and purification of these proteins and screening of these potential vaccine candidates are needed.

## Author contributions

Conceived and design the experiment: YZZ, XG, Y-FC, and YY; compartive genomic analysis: LZ and DW; predicting subcellular localization: LZ, NH, QZ, and KC; screening known surface-exposed antigens: KD and YZ; write the manuscript: LZ, XG, YZZ, Y-FC, and YY.

### Conflict of interest statement

The authors declare that the research was conducted in the absence of any commercial or financial relationships that could be construed as a potential conflict of interest.

## Supplementary material

Table S1 **All of core, dispensable and specific PSEs among the 17 representative strains of pathogenic *L. interrogans***.

Table S2 **Experimentally confirmed surface-exposed antigens or virulence factors of pathogenic *Leptospira***.

Table S3 **The detailed known antigens among Hakke's result, our result and Yang's result**.
