# Supplementary material for: A Novel Pan-Genome Reverse Vaccinology Approach Employing a Negative-Selection Strategy for Screening Surface-Exposed Antigens against leptospirosis
Source: Front Microbiol. 2017 Mar 14;8:396. doi: 10.3389/fmicb.2017.00396 (PMC5348505; doi:10.3389/fmicb.2017.00396)
Supplement: Table S2 — Experimentally confirmed surface-exposed antigens or virulence factors of pathogenic Leptospira. [file Table2.DOCX]

| **Locus** | **Label** | **Annotation** | **Function** | **References** |
| --- | --- | --- | --- | --- |
| LIC12906/LA0695 | LenA/Lsa24/LfhA | putative lipoprotein | surface adhesin binding to laminin and Factor H | ([Barbosa et al., 2006](#_ENREF_4)) |
| LIC10997/LA3103 | LenB | Endostatin-like protein | surface adhesin binding to Fibronectin/Laminin | ([Stevenson et al., 2007](#_ENREF_42)) |
| LIC13006/LA0563 | LenC | Endostatin-like protein | surface adhesin binding to Fibronectin/Laminin | ([Stevenson et al., 2007](#_ENREF_42)) |
| LIC12315/LA1433 | LenD | Endostatin-like protein | surface adhesin binding to Fibronectin/Laminin | ([Stevenson et al., 2007](#_ENREF_42)) |
| LIC13467/LA4324 | LenE | Endostatin-like protein | surface adhesin binding to Fibronectin/Laminin | ([Stevenson et al., 2007](#_ENREF_42)) |
| LIC13248/LA4073 | LenF | Endostatin-like protein | surface adhesin binding to Fibronectin/Laminin | ([Stevenson et al., 2007](#_ENREF_42)) |
| LIC10011/LA0011 | LipL21 | outer membrane lipoprotein | important antigen | ([Lin et al., 2010](#_ENREF_23)) |
| LIC11352/LA2637 | LipL32 | outer membrane lipoprotein | surface adhesin binding to Laminin/Collagen I/Collagen V/Collagen IV/Fibronectin | ([Hauk et al., 2008](#_ENREF_18);[Hoke et al., 2008](#_ENREF_19)) |
| LIC13060/LA0492 | LipL36 | outer membrane lipoprotein | known antigen | ([Haake et al., 1998](#_ENREF_15)) |
| LIC10091/LA0103 | LipL40 | Putative lipoprotein | surface adhesin binding to plasminogen | ([Gamberini et al., 2005](#_ENREF_13);[Vieira et al., 2010](#_ENREF_47)) |
| LIC12966/LA0616 | LipL41 | Putative lipoprotein | known antigens | ([Asuthkar et al., 2007](#_ENREF_1);[Lin et al., 2011](#_ENREF_22)) |
| LIC11885/LA2024 | LipL46 | Putative lipoprotein | major antigens | ([Matsunaga et al., 2006](#_ENREF_27)) |
| LIC10898/LA3240 | LipL48 | putative outer membrane lipoprotein | major OMP | ([Haake and Matsunaga, 2002](#_ENREF_16);[Xue et al., 2010](#_ENREF_50)) |
| LIC12099/LA1691 | LipL53 | hypothetical protein | surface adhesin binding to Collagen IV/Laminin/Fibronectin | ([Oliveira et al., 2010](#_ENREF_35)) |
| LIC10465/LA3778 | LigA | LigA-like protein | surface adhesin binding to Collagen I/Collagen IV/Laminin/Fibronectin/Tropoelastin | ([Choy et al., 2007](#_ENREF_8);[Lin et al., 2009](#_ENREF_24)) |
| LIC10464/LA3075 | LigB | LigB-like protein | surface adhesin binding to Collagen I/Collagen IV/Collagen III/Laminin/Fibronectin/Elastin/Tropoelastin/Heparin | ([Choy et al., 2007](#_ENREF_8);[Lin et al., 2009](#_ENREF_24);[Choy et al., 2011](#_ENREF_9);[Ching et al., 2012](#_ENREF_7)) |
| LIC11003/LA3097 | LruA | lipoprotein | immunopathogenic factor | ([Verma et al., 2005](#_ENREF_45)) |
| LIC10713/LA3469 | LruB/MFn14 | lipoprotein | immunopathogenic factor | ([Verma et al., 2005](#_ENREF_45)) |
| LIC20172/LB217 | LruC | lipoprotein | pathogenic factor | ([Verma et al., 2005](#_ENREF_45)) |
| LIC11947/LA1957 | LcpA | Leptospiral complement regulator-acquiring protein | surface adhesin binding to complement regulator C4BP | ([Barbosa et al., 2010](#_ENREF_5)) |
| LIC11058/LA3017 | LemA | putative lipoprotein | subunit vaccine antigen | ([Hartwig et al., 2013](#_ENREF_17)) |
| LIC11469/LA2469 | Lsa20 | putative lipoprotein | surface adhesin binding laminin and human plasminogen | ([Mendes et al., 2011](#_ENREF_28)) |
| LIC10368/LA0419 | Lsa21 | putative lipoprotein | surface adhesin binding to Collagen IV/Laminin/Fibronectin | ([Atzingen et al., 2008](#_ENREF_2)) |
| LIC11360/LA2626 | Lsa23 | putative lipoprotein | surface adhesin binding to Fibrinogen | ([Siqueira et al., 2016](#_ENREF_39)) |
| LIC12253/LA1508 | Lsa25 | putative lipoprotein | surface adhesin binding to Laminin | ([Domingos et al., 2012](#_ENREF_10)) |
| LIC12895/LA0710 | Lsa27 | putative lipoprotein | surface adhesin binding to Laminin | ([Vieira et al., 2014](#_ENREF_48)) |
| LIC11087/LA2975 | Lsa30 | putative lipoprotein | surface adhesin binding to Laminin/Fibronectin | ([Souza et al., 2012](#_ENREF_40)) |
| LIC11834/LA2083 | Lsa33 | putative lipoprotein | surface adhesin binding to Laminin | ([Domingos et al., 2012](#_ENREF_10)) |
| LIC11975/LA1931 | Lsa36 | outer membrane protein | surface adhesin binding to Fibrinogen | ([Oliveira et al., 2013](#_ENREF_33)) |
| LIC13479/LA4337 | Lsa46 | OmpA family protein | surface adhesin binding Laminin | ([Teixeira et al., 2015](#_ENREF_43)) |
| LIC10314/LA0365 | Lsa63 | putative lipoprotein | surface adhesin binding to Collagen IV/Laminin | ([Vieira et al., 2010](#_ENREF_47)) |
| LIC10258/LA0301 | Lsa66/MFn8 | putative lipoprotein | surface adhesin binding to Laminin/Fibronectin | ([Pinne et al., 2012](#_ENREF_37)) |
| LIC10050/LA0056 | Lsa77 | OmpA family protein | surface adhesin binding Laminin | ([Teixeira et al., 2015](#_ENREF_43)) |
| LIC12892/LA0532 | Lp29 | putative lipoprotein | surface antigen binding plasminogen | ([Neves et al., 2007](#_ENREF_30);[Vieira et al., 2010](#_ENREF_47)) |
| LIC12880/LA0730 | Lp30 | Hypothetical protein | surface adhesin binding to plasminogen | ([Oliveira et al., 2011](#_ENREF_32)) |
| LIC10793/LA3370 | Lp49 | surface antigen OrfC lipoprotein | serodiagnostic antigen | ([Neves et al., 2007](#_ENREF_30)) |
| LIC12690/LA0962 | Lp95 | putative lipoprotein | surface adhesin binding to Laminin/Fibronectin | ([Atzingen et al., 2009](#_ENREF_3)) |
| LIC10973/LA3138 | OmpL1 | outer membrane protein | surface adhesin binding to Laminin/Fibronectin | ([Haake et al., 1993](#_ENREF_14);[Pinne and Haake, 2009](#_ENREF_36);[Fernandes et al., 2012](#_ENREF_12)) |
| LIC11848/LA2066 | OmpL32 | hypothetical protein | virulecne-associated OMP | ([Eshghi et al., 2012](#_ENREF_11)) |
| LIC13166/LA3961 | OmpL36 | conserved hypothetical protein | surface-exposed OMP | ([Pinne and Haake, 2009](#_ENREF_36)) |
| LIC12263/LA1495 | OmpL37 | conserved hypothetical protein | surface adhesin binding to Laminin/Fibronectin/Elastin | ([Pinne and Haake, 2009](#_ENREF_36)) |
| LIC13050/LA0505 | OmpL47 | conserved hypothetical protein | surface adhesin binding to Collagen III/Laminin/Fibronectin/Elastin | ([Pinne and Haake, 2009](#_ENREF_36)) |
| LIC10592/LA3615 | Omp52 | OmpA family protein | interaction of host cells | ([Hsieh et al., 2005](#_ENREF_20)) |
| LIC13491/LA4349 | OmpL54 | metallopeptidase | surface-exposed OMP | ([Pinne and Haake, 2009](#_ENREF_36)) |
| LIC11612/LA2330 | MFn1 | Hypothetical protein | surface adhesin binding to Fibronectin | ([Pinne et al., 2012](#_ENREF_37)) |
| LIC10714/LA3468 | MFn2 | TonB-dependent receptor | surface adhesin binding to Fibronectin | ([Pinne et al., 2012](#_ENREF_37)) |
| LIC13198/LA4004 | MFn3/Sph3 | Sphingomyelinase 3 | surface-exposed protein | ([Pinne et al., 2012](#_ENREF_37)) |
| LIC12631/LA1029 | MFn4/Sph2 | Sphingomyelinase 2 | Mg(++)-dependent hemolysin | ([Narayanavari et al., 2012](#_ENREF_29);[Pinne et al., 2012](#_ENREF_37)) |
| LIC13135/LA3927 | MFn5/TolC | outer membrane family protein | surface adhesin binding to Fibronectin | ([Pinne et al., 2012](#_ENREF_37)) |
| LIC11051/LA3028 | MFn6 | conserved hypothetical protein | surface adhesin binding to Fibronectin | ([Pinne et al., 2012](#_ENREF_37)) |
| LIC11436/LA2537 | MFn7 | conserved hypothetical protein | surface adhesin binding to Fibronectin | ([Pinne et al., 2012](#_ENREF_37)) |
| LIC10537/LA3685 | MFn9 | OmpA-related protein | surface adhesin binding to Fibronectin | ([Pinne et al., 2012](#_ENREF_37)) |
| LIC11755/LA2167 | MFn10 | Hypothetical protein | surface adhesin binding to Fibronectin | ([Pinne et al., 2012](#_ENREF_37)) |
| LIC11028/LA3067 | MFn11 | Tol transport system component | surface-exposed OMP | ([Pinne et al., 2012](#_ENREF_37)) |
| LIC12952/LA0635 | MFn12 | Hypothetical protein | surface-exposed OMP | ([Pinne et al., 2012](#_ENREF_37)) |
| LIC11893/LA2014 | MFn13 | CreD-like protein | surface-exposed OMP | ([Pinne et al., 2012](#_ENREF_37)) |
| LIC13066/LA3834 | MFn15 | Hypothetical protein | surface-exposed OMP | ([Pinne et al., 2012](#_ENREF_37)) |
| LIC20151/LB191 | HbpA | TonB-dependent hemin-binding protein | Fe3+-siderophores/hemin receptors | ([Asuthkar et al., 2007](#_ENREF_1)) |
| LIC12238/LA1523 |  | conserved hypothetical protein | surface adhesin binding to plasminogen | ([Vieira et al., 2010](#_ENREF_47);[Oliveira et al., 2011](#_ENREF_32);[Oliveira et al., 2013](#_ENREF_33)) |
| LIC10494/LA3735 |  | putative lipoprotein | surface adhesin binding to plasminogen | ([Vieira et al., 2010](#_ENREF_47)) |
| LIC11360/LA2626 |  | putative lipoprotein | surface adhesin binding to fibrinogen | ([Oliveira et al., 2013](#_ENREF_33)) |
| LIC12730/LA0913 |  | conserved hypothetical protein | surface adhesin binding to plasminogen | ([Verma et al., 2012](#_ENREF_46)) |
| LIC13143/LA3937 | TlyC | hemolysin | surface adhesin binding to Collagen IV/Laminin/Fibronectin | ([Carvalho et al., 2009](#_ENREF_6)) |
| LIC11859/LA2055 | Mce | MCE-like protein | surface adhesin binding to integrins α5β1 and α(V) β3 | ([Zhang et al., 2012](#_ENREF_51)) |
| LIC10714/LA3468 | FecA | TonB-dependent receptor | confirmed OMP | ([Louvel et al., 2005](#_ENREF_26)) |
| LIC10191/LA0222 | Loa22 | OmpA family lipoprotein | recognized virulence factor | ([Ristow et al., 2007](#_ENREF_38)) |
| LIC10765/LA3405 | MPL17 | conserved hypothetical protein | serodiagnotic antigen | ([Oliveira et al., 2008](#_ENREF_34)) |
| LIC13131/LA3922 | MPL21 | conserved hypothetical protein | serodiagnotic antigen | ([Oliveira et al., 2008](#_ENREF_34)) |
| LIC10054/LA0061 | MPL36 | RlpA-like lipoprotein | surface adhesin binding to Plasminogen | ([Vieira et al., 2010](#_ENREF_47)) |
| LIC12875/LA0737 | EF-Tu | elongation factor Tu | surface adhesin binding to plasminogen | ([Wolff et al., 2013](#_ENREF_49)) |
| LIC12976/LA0602 |  | NAD/FAD-binding protein | surface adhesin binding to Laminin | ([Lima et al., 2013](#_ENREF_21)) |
| LIC20172/LB216 | LMB216 | lipoprotein | essential virulence factor | ([Toma et al., 2014](#_ENREF_44)) |
| LIC11954/LA1951 |  | enolase | surface adhesin binding to plasminogen | ([Nogueira et al., 2013](#_ENREF_31)) |
| LIC11573/LA2372 | gspG | type II secretory pathway component protein G | protective antigens | ([Srikram et al., 2011](#_ENREF_41)) |
| LIC12085/LA1939 |  | hypothetical protein | protective antigens | ([Srikram et al., 2011](#_ENREF_41)) |
| LIC11694/LA2242 | CirA | TonB-dependent outer membrane receptor | confirmed OMP | ([Louvel et al., 2006](#_ENREF_25)) |
| LIC12998/LA0572 |  | TonB-dependent outer membrane receptor | OMP | ([Louvel et al., 2006](#_ENREF_25)) |
| LIC12898/LA0706 |  | TonB-dependent outer membrane receptor | OMP | ([Louvel et al., 2006](#_ENREF_25)) |
| LIC12374/LA1356 |  | TonB-dependent outer membrane receptor | OMP | ([Louvel et al., 2006](#_ENREF_25)) |
| LIC10964/LA3149 |  | TonB-dependent outer membrane receptor | OMP | ([Louvel et al., 2006](#_ENREF_25)) |
| LIC11345/LA2641 |  | TonB-dependent outer membrane receptor | OMP | ([Louvel et al., 2006](#_ENREF_25)) |

Asuthkar, S., Velineni, S., Stadlmann, J., Altmann, F., and Sritharan, M. (2007). Expression and characterization of an iron-regulated hemin-binding protein, HbpA, from Leptospira interrogans serovar Lai. *Infect Immun* 75**,** 4582-4591. doi: 10.1128/IAI.00324-07.

Atzingen, M.V., Barbosa, A.S., De Brito, T., Vasconcellos, S.A., de Morais, Z.M., Lima, D.M., Abreu, P.A., and Nascimento, A.L. (2008). Lsa21, a novel leptospiral protein binding adhesive matrix molecules and present during human infection. *BMC Microbiol* 8**,** 70. doi: 10.1186/1471-2180-8-70.

Atzingen, M.V., Gomez, R.M., Schattner, M., Pretre, G., Goncales, A.P., de Morais, Z.M., Vasconcellos, S.A., and Nascimento, A.L. (2009). Lp95, a novel leptospiral protein that binds extracellular matrix components and activates e-selectin on endothelial cells. *J Infect* 59**,** 264-276. doi: 10.1016/j.jinf.2009.07.010.

Barbosa, A.S., Abreu, P.A., Neves, F.O., Atzingen, M.V., Watanabe, M.M., Vieira, M.L., Morais, Z.M., Vasconcellos, S.A., and Nascimento, A.L. (2006). A newly identified leptospiral adhesin mediates attachment to laminin. *Infect Immun* 74**,** 6356-6364. doi: 10.1128/IAI.00460-06.

Barbosa, A.S., Monaris, D., Silva, L.B., Morais, Z.M., Vasconcellos, S.A., Cianciarullo, A.M., Isaac, L., and Abreu, P.A. (2010). Functional characterization of LcpA, a surface-exposed protein of Leptospira spp. that binds the human complement regulator C4BP. *Infect Immun* 78**,** 3207-3216. doi: 10.1128/IAI.00279-10.

Carvalho, E., Barbosa, A.S., Gomez, R.M., Cianciarullo, A.M., Hauk, P., Abreu, P.A., Fiorini, L.C., Oliveira, M.L., Romero, E.C., Goncales, A.P., Morais, Z.M., Vasconcellos, S.A., and Ho, P.L. (2009). Leptospiral TlyC is an extracellular matrix-binding protein and does not present hemolysin activity. *FEBS Lett* 583**,** 1381-1385. doi: 10.1016/j.febslet.2009.03.050.

Ching, A.T., Favaro, R.D., Lima, S.S., Chaves Ade, A., de Lima, M.A., Nader, H.B., Abreu, P.A., and Ho, P.L. (2012). Leptospira interrogans shotgun phage display identified LigB as a heparin-binding protein. *Biochem Biophys Res Commun* 427**,** 774-779. doi: 10.1016/j.bbrc.2012.09.137.

Choy, H.A., Kelley, M.M., Chen, T.L., Moller, A.K., Matsunaga, J., and Haake, D.A. (2007). Physiological osmotic induction of Leptospira interrogans adhesion: LigA and LigB bind extracellular matrix proteins and fibrinogen. *Infect Immun* 75**,** 2441-2450. doi: 10.1128/IAI.01635-06.

Choy, H.A., Kelley, M.M., Croda, J., Matsunaga, J., Babbitt, J.T., Ko, A.I., Picardeau, M., and Haake, D.A. (2011). The multifunctional LigB adhesin binds homeostatic proteins with potential roles in cutaneous infection by pathogenic Leptospira interrogans. *PLoS One* 6**,** e16879. doi: 10.1371/journal.pone.0016879.

Domingos, R.F., Vieira, M.L., Romero, E.C., Goncales, A.P., de Morais, Z.M., Vasconcellos, S.A., and Nascimento, A.L. (2012). Features of two proteins of Leptospira interrogans with potential role in host-pathogen interactions. *BMC Microbiol* 12**,** 50. doi: 10.1186/1471-2180-12-50.

Eshghi, A., Lourdault, K., Murray, G.L., Bartpho, T., Sermswan, R.W., Picardeau, M., Adler, B., Snarr, B., Zuerner, R.L., and Cameron, C.E. (2012). Leptospira interrogans catalase is required for resistance to H2O2 and for virulence. *Infect Immun* 80**,** 3892-3899. doi: 10.1128/IAI.00466-12.

Fernandes, L.G., Vieira, M.L., Kirchgatter, K., Alves, I.J., de Morais, Z.M., Vasconcellos, S.A., Romero, E.C., and Nascimento, A.L. (2012). OmpL1 is an extracellular matrix- and plasminogen-interacting protein of Leptospira spp. *Infect Immun* 80**,** 3679-3692. doi: 10.1128/IAI.00474-12.

Gamberini, M., Gomez, R.M., Atzingen, M.V., Martins, E.A., Vasconcellos, S.A., Romero, E.C., Leite, L.C., Ho, P.L., and Nascimento, A.L. (2005). Whole-genome analysis of Leptospira interrogans to identify potential vaccine candidates against leptospirosis. *FEMS Microbiol Lett* 244**,** 305-313. doi: 10.1016/j.femsle.2005.02.004.

Haake, D.A., Champion, C.I., Martinich, C., Shang, E.S., Blanco, D.R., Miller, J.N., and Lovett, M.A. (1993). Molecular cloning and sequence analysis of the gene encoding OmpL1, a transmembrane outer membrane protein of pathogenic Leptospira spp. *J Bacteriol* 175**,** 4225-4234.

Haake, D.A., Martinich, C., Summers, T.A., Shang, E.S., Pruetz, J.D., McCoy, A.M., Mazel, M.K., and Bolin, C.A. (1998). Characterization of leptospiral outer membrane lipoprotein LipL36: downregulation associated with late-log-phase growth and mammalian infection. *Infect Immun* 66**,** 1579-1587.

Haake, D.A., and Matsunaga, J. (2002). Characterization of the leptospiral outer membrane and description of three novel leptospiral membrane proteins. *Infect Immun* 70**,** 4936-4945.

Hartwig, D.D., Forster, K.M., Oliveira, T.L., Amaral, M., McBride, A.J., and Dellagostin, O.A. (2013). A prime-boost strategy using the novel vaccine candidate, LemA, protects hamsters against leptospirosis. *Clin Vaccine Immunol* 20**,** 747-752. doi: 10.1128/CVI.00034-13.

Hauk, P., Macedo, F., Romero, E.C., Vasconcellos, S.A., de Morais, Z.M., Barbosa, A.S., and Ho, P.L. (2008). In LipL32, the major leptospiral lipoprotein, the C terminus is the primary immunogenic domain and mediates interaction with collagen IV and plasma fibronectin. *Infect Immun* 76**,** 2642-2650. doi: 10.1128/IAI.01639-07.

Hoke, D.E., Egan, S., Cullen, P.A., and Adler, B. (2008). LipL32 is an extracellular matrix-interacting protein of Leptospira spp. and Pseudoalteromonas tunicata. *Infect Immun* 76**,** 2063-2069. doi: 10.1128/IAI.01643-07.

Hsieh, W.J., Chang, Y.F., Chen, C.S., and Pan, M.J. (2005). Omp52 is a growth-phase-regulated outer membrane protein of Leptospira santarosai serovar Shermani. *FEMS Microbiol Lett* 243**,** 339-345. doi: 10.1016/j.femsle.2004.12.021.

Lima, S.S., Ching, A.T., Favaro, R.D., Da Silva, J.B., Oliveira, M.L., Carvalho, E., Abreu, P.A., Vasconcellos, S.A., and Ho, P.L. (2013). Adhesin activity of Leptospira interrogans lipoprotein identified by in vivo and in vitro shotgun phage display. *Biochem Biophys Res Commun* 431**,** 342-347. doi: 10.1016/j.bbrc.2012.12.095.

Lin, X., Sun, A., Ruan, P., Zhang, Z., and Yan, J. (2011). Characterization of conserved combined T and B cell epitopes in Leptospira interrogans major outer membrane proteins OmpL1 and LipL41. *BMC Microbiol* 11**,** 21. doi: 10.1186/1471-2180-11-21.

Lin, X., Zhao, J., Qian, J., Mao, Y., Pan, J., Li, L., Peng, H., Luo, Y., and Yan, J. (2010). Identification of immunodominant B- and T-cell combined epitopes in outer membrane lipoproteins LipL32 and LipL21 of Leptospira interrogans. *Clin Vaccine Immunol* 17**,** 778-783. doi: 10.1128/CVI.00405-09.

Lin, Y.P., Greenwood, A., Yan, W., Nicholson, L.K., Sharma, Y., McDonough, S.P., and Chang, Y.F. (2009). A novel fibronectin type III module binding motif identified on C-terminus of Leptospira immunoglobulin-like protein, LigB. *Biochem Biophys Res Commun* 389**,** 57-62. doi: 10.1016/j.bbrc.2009.08.089.

Louvel, H., Bommezzadri, S., Zidane, N., Boursaux-Eude, C., Creno, S., Magnier, A., Rouy, Z., Medigue, C., Saint Girons, I., Bouchier, C., and Picardeau, M. (2006). Comparative and functional genomic analyses of iron transport and regulation in Leptospira spp. *J Bacteriol* 188**,** 7893-7904. doi: 10.1128/JB.00711-06.

Louvel, H., Saint Girons, I., and Picardeau, M. (2005). Isolation and characterization of FecA- and FeoB-mediated iron acquisition systems of the spirochete Leptospira biflexa by random insertional mutagenesis. *J Bacteriol* 187**,** 3249-3254. doi: 10.1128/JB.187.9.3249-3254.2005.

Matsunaga, J., Werneid, K., Zuerner, R.L., Frank, A., and Haake, D.A. (2006). LipL46 is a novel surface-exposed lipoprotein expressed during leptospiral dissemination in the mammalian host. *Microbiology* 152**,** 3777-3786. doi: 10.1099/mic.0.29162-0.

Mendes, R.S., Von Atzingen, M., de Morais, Z.M., Goncales, A.P., Serrano, S.M., Asega, A.F., Romero, E.C., Vasconcellos, S.A., and Nascimento, A.L. (2011). The novel leptospiral surface adhesin Lsa20 binds laminin and human plasminogen and is probably expressed during infection. *Infect Immun* 79**,** 4657-4667. doi: 10.1128/IAI.05583-11.

Narayanavari, S.A., Kishore, N.M., and Sritharan, M. (2012). Structural analysis of the Leptospiral sphingomyelinases: in silico and experimental evaluation of Sph2 as an Mg-dependent sphingomyelinase. *J Mol Microbiol Biotechnol* 22**,** 24-34. doi: 10.1159/000337013.

Neves, F.O., Abreu, P.A., Vasconcellos, S.A., de Morais, Z.M., Romero, E.C., and Nascimento, A.L. (2007). Identification of a novel potential antigen for early-phase serodiagnosis of leptospirosis. *Arch Microbiol* 188**,** 523-532. doi: 10.1007/s00203-007-0273-2.

Nogueira, S.V., Backstedt, B.T., Smith, A.A., Qin, J.H., Wunder, E.A., Jr., Ko, A., and Pal, U. (2013). Leptospira interrogans enolase is secreted extracellularly and interacts with plasminogen. *PLoS One* 8**,** e78150. doi: 10.1371/journal.pone.0078150.

Oliveira, R., de Morais, Z.M., Goncales, A.P., Romero, E.C., Vasconcellos, S.A., and Nascimento, A.L. (2011). Characterization of novel OmpA-like protein of Leptospira interrogans that binds extracellular matrix molecules and plasminogen. *PLoS One* 6**,** e21962. doi: 10.1371/journal.pone.0021962.

Oliveira, R., Domingos, R.F., Siqueira, G.H., Fernandes, L.G., Souza, N.M., Vieira, M.L., de Morais, Z.M., Vasconcellos, S.A., and Nascimento, A.L. (2013). Adhesins of Leptospira interrogans mediate the interaction to fibrinogen and inhibit fibrin clot formation in vitro. *PLoS Negl Trop Dis* 7**,** e2396. doi: 10.1371/journal.pntd.0002396.

Oliveira, T.R., Longhi, M.T., de Morais, Z.M., Romero, E.C., Blanco, R.M., Kirchgatter, K., Vasconcellos, S.A., and Nascimento, A.L. (2008). Evaluation of leptospiral recombinant antigens MPL17 and MPL21 for serological diagnosis of leptospirosis by enzyme-linked immunosorbent assays. *Clin Vaccine Immunol* 15**,** 1715-1722. doi: 10.1128/CVI.00214-08.

Oliveira, T.R., Longhi, M.T., Goncales, A.P., de Morais, Z.M., Vasconcellos, S.A., and Nascimento, A.L. (2010). LipL53, a temperature regulated protein from Leptospira interrogans that binds to extracellular matrix molecules. *Microbes Infect* 12**,** 207-217. doi: 10.1016/j.micinf.2009.12.004.

Pinne, M., and Haake, D.A. (2009). A comprehensive approach to identification of surface-exposed, outer membrane-spanning proteins of Leptospira interrogans. *PLoS One* 4**,** e6071. doi: 10.1371/journal.pone.0006071.

Pinne, M., Matsunaga, J., and Haake, D.A. (2012). Leptospiral outer membrane protein microarray, a novel approach to identification of host ligand-binding proteins. *J Bacteriol* 194**,** 6074-6087. doi: 10.1128/JB.01119-12.

Ristow, P., Bourhy, P., da Cruz McBride, F.W., Figueira, C.P., Huerre, M., Ave, P., Girons, I.S., Ko, A.I., and Picardeau, M. (2007). The OmpA-like protein Loa22 is essential for leptospiral virulence. *PLoS Pathog* 3**,** e97. doi: 10.1371/journal.ppat.0030097.

Siqueira, G.H., Atzingen, M.V., de Souza, G.O., Vasconcellos, S.A., and Nascimento, A.L. (2016). Leptospira interrogans Lsa23 protein recruits plasminogen, factor H and C4BP from normal human serum and mediates C3b and C4b degradation. *Microbiology* 162**,** 295-308. doi: 10.1099/mic.0.000217.

Souza, N.M., Vieira, M.L., Alves, I.J., de Morais, Z.M., Vasconcellos, S.A., and Nascimento, A.L. (2012). Lsa30, a novel adhesin of Leptospira interrogans binds human plasminogen and the complement regulator C4bp. *Microb Pathog* 53**,** 125-134. doi: 10.1016/j.micpath.2012.06.001.

Srikram, A., Zhang, K., Bartpho, T., Lo, M., Hoke, D.E., Sermswan, R.W., Adler, B., and Murray, G.L. (2011). Cross-protective immunity against leptospirosis elicited by a live, attenuated lipopolysaccharide mutant. *J Infect Dis* 203**,** 870-879. doi: 10.1093/infdis/jiq127.

Stevenson, B., Choy, H.A., Pinne, M., Rotondi, M.L., Miller, M.C., Demoll, E., Kraiczy, P., Cooley, A.E., Creamer, T.P., Suchard, M.A., Brissette, C.A., Verma, A., and Haake, D.A. (2007). Leptospira interrogans endostatin-like outer membrane proteins bind host fibronectin, laminin and regulators of complement. *PLoS One* 2**,** e1188. doi: 10.1371/journal.pone.0001188.

Teixeira, A.F., de Morais, Z.M., Kirchgatter, K., Romero, E.C., Vasconcellos, S.A., and Nascimento, A.L. (2015). Features of two new proteins with OmpA-like domains identified in the genome sequences of Leptospira interrogans. *PLoS One* 10**,** e0122762. doi: 10.1371/journal.pone.0122762.

Toma, C., Murray, G.L., Nohara, T., Mizuyama, M., Koizumi, N., Adler, B., and Suzuki, T. (2014). Leptospiral outer membrane protein LMB216 is involved in enhancement of phagocytic uptake by macrophages. *Cell Microbiol* 16**,** 1366-1377. doi: 10.1111/cmi.12296.

Verma, A., Artiushin, S., Matsunaga, J., Haake, D.A., and Timoney, J.F. (2005). LruA and LruB, novel lipoproteins of pathogenic Leptospira interrogans associated with equine recurrent uveitis. *Infect Immun* 73**,** 7259-7266. doi: 10.1128/IAI.73.11.7259-7266.2005.

Verma, A., Matsunaga, J., Artiushin, S., Pinne, M., Houwers, D.J., Haake, D.A., Stevenson, B., and Timoney, J.F. (2012). Antibodies to a novel leptospiral protein, LruC, in the eye fluids and sera of horses with Leptospira-associated uveitis. *Clin Vaccine Immunol* 19**,** 452-456. doi: 10.1128/CVI.05524-11.

Vieira, M.L., Atzingen, M.V., Oliveira, T.R., Oliveira, R., Andrade, D.M., Vasconcellos, S.A., and Nascimento, A.L. (2010). In vitro identification of novel plasminogen-binding receptors of the pathogen Leptospira interrogans. *PLoS One* 5**,** e11259. doi: 10.1371/journal.pone.0011259.

Vieira, M.L., Fernandes, L.G., Domingos, R.F., Oliveira, R., Siqueira, G.H., Souza, N.M., Teixeira, A.R., Atzingen, M.V., and Nascimento, A.L. (2014). Leptospiral extracellular matrix adhesins as mediators of pathogen-host interactions. *FEMS Microbiol Lett* 352**,** 129-139. doi: 10.1111/1574-6968.12349.

Wolff, D.G., Castiblanco-Valencia, M.M., Abe, C.M., Monaris, D., Morais, Z.M., Souza, G.O., Vasconcellos, S.A., Isaac, L., Abreu, P.A., and Barbosa, A.S. (2013). Interaction of Leptospira elongation factor Tu with plasminogen and complement factor H: a metabolic leptospiral protein with moonlighting activities. *PLoS One* 8**,** e81818. doi: 10.1371/journal.pone.0081818.

Xue, F., Dong, H., Wu, J., Wu, Z., Hu, W., Sun, A., Troxell, B., Yang, X.F., and Yan, J. (2010). Transcriptional responses of Leptospira interrogans to host innate immunity: significant changes in metabolism, oxygen tolerance, and outer membrane. *PLoS Negl Trop Dis* 4**,** e857. doi: 10.1371/journal.pntd.0000857.

Zhang, L., Zhang, C., Ojcius, D.M., Sun, D., Zhao, J., Lin, X., Li, L., Li, L., and Yan, J. (2012). The mammalian cell entry (Mce) protein of pathogenic Leptospira species is responsible for RGD motif-dependent infection of cells and animals. *Mol Microbiol* 83**,** 1006-1023. doi: 10.1111/j.1365-2958.2012.07985.x.
